# Next Generation Weight Loss Drugs for the Prevention of Cancer?

**DOI:** 10.1177/10732748241241158

**Published:** 2024-03-22

**Authors:** Chantelle Carbonell, Mariet Mathew Stephen, Yibing Ruan, Matthew T. Warkentin, Darren R. Brenner

**Affiliations:** 1 Department of Oncology, Cumming School of Medicine, 213572 University of Calgary, Calgary, AB, Canada; 2 Department of Cancer Epidemiology and Prevention Research, Cancer Care Alberta, Alberta Health Services, Calgary, AB, Canada; 3 Department of Community Health Sciences, Cumming School of Medicine, 213572 University of Calgary, Calgary, AB, Canada

**Keywords:** cancer prevention, pharmacology, chemoprevention, Glucagon-like peptide-1 receptor agonists, weight loss

## Abstract

**Background:** Western populations are losing the battle over healthy weight management, and excess body weight is a notable cancer risk factor at the population level. There is ongoing interest in pharmacological interventions aimed at promoting weight loss, including GLP-1 receptor agonists (GLP-1RA), which may be a useful tool to stem the rising tide of obesity-related cancers.

**Purpose:** To investigate the potential of next generation weight loss drugs (NGWLD) like GLP-1RA in population-level chemoprevention.

**Research Design:** We used the OncoSim microsimulation tool to estimate the population-level reductions in obesity and the potentially avoidable obesity-related cancers in Canada over the next 25 years.

**Results:** We estimated a total of 71 281 preventable cancers by 2049, with 36 235 and 35 046 cancers prevented for females and males, respectively. Among the 327 254 total projected cancer cases in 2049, 1.3% are estimated to be preventable through intervention with NGWLD.

**Conclusions:** Pharmacologic intervention is not the ideal solution for the obesity-related cancer crisis. However, these agents and subsequent generations provide an additional tool to rapidly reduce body weight and adiposity in populations that have been extremely challenging to reduce weight with standard diet and exercise approaches. Additional research is needed around approaches to prevent initial weight gain and maintain long-term weight loss.

## Introduction

Western countries are losing the battle over healthy weight management. Modern diets, coupled with changes to behaviour and activity patterns, have created a considerable challenge for people to maintain healthy body weights. Energy dense, nutrient poor diets have dramatically increased the average caloric intake with foods that are designed primarily for shelf stability and flavour rather than dietary quality. Urban development and modern employment trends have lowered the amount of physical activity in adults and digital technology has done the same among younger age groups.^1,2^ Increased body weight considerably elevates the risk of nearly all chronic diseases, including thirteen cancer types.^3^ Many cancer types associated with excess body weight have been increasing and expected to continue over the next 20 years. Cancer rates are also on the rise among younger individuals, specifically those aged 40-49 years, with statistically meaningful increases in recent generations for common cancers linked to obesity such as colorectal and breast.^4,5^ Despite ongoing awareness-based prevention activities, their impact on controlling the weight at the population-level has been minimal.

### Increasing Body Size and Cancer Rates

According to data from the 2017-2018 National Health and Nutrition Examination Survey (NHANES), 31% of U.S. adults aged 20 and older have a body mass index (BMI) that classifies them as overweight (BMI 25.0-29.9), and an additional 42% as obese (BMI equal to or greater than 30).^6^ Similarly, in 2018, 36% and 27% of Canadian adults aged 18 and older reported BMIs that classifies them as overweight and obese, respectively.^7^ Furthermore – those with class II (BMI 35.0-39.9) and III obesity (BMI 40.0-49.9) are increasing across all age groups.^8^ It has been well established for almost 20 years that excess body mass is a meaningful driver of cancer risk at the population level.^9-11^ During this time, the average BMI has continued to increase.^10^ As noted – Western populations are reporting a rise in common cancers related to excess adiposity, including colorectal, breast (postmenopausal), endometrial and many others.^11^

### Limited Success to Date

Despite broad awareness of the importance of healthy weight management, few solutions have proven successful in achieving long-term weight reduction. Weight management over the life course is a lifelong challenge that requires continual attention. Many weight loss interventions, including lifestyle, activity and diet modifications, have yielded less than impressive results in terms of long-term weight reduction.^12^ Lack of motivation and time, societal pressures, and physical limitations are commonly reported barriers to weight loss efforts.^12^ Socioeconomic status is also an important factor in determining access to affordable facilities, physical training classes and memberships, and nutrient-dense food options.^12^ Often excess body size is an equity issue as quality foods and activity programs can be costly over the long term.

### Pharmacotherapy as a Potential Breakthrough for Cancer Prevention?

There is ongoing interest in the field of pharmacological interventions aimed at promoting weight loss. Given the extremely high prevalence of metabolic syndrome and type II diabetes in Western populations, several different agents have been evaluated to manage hemoglobin A1c (HbA1c) levels and potentially impact weight loss. Glucagon-like peptide-1 (GLP-1) is an endogenous hormone released by the gastrointestinal tract in response to food intake and binds to the GLP-1 receptor.^13^ GLP-1 receptor agonists (GLP-1RA) act in several places including the brain to reduce food intake and modulate diabetes.^13^ Recent data from multiple phase III trials of GLP-1-RA among adults with cardiometabolic risk factors and excess body weight have been impressive.^14-20^ A summary of the recent phase III trial results is included in Table 1. The trials presented herein included individuals aged 18 years or older with a BMI of ≥30 and individuals with a BMI of ≥27 with a comorbid condition.^14-20^ In most cases, comorbid conditions included very common cardiometabolic measures such as elevated blood pressure or dyslipidemia.^14-17,20^ Perhaps most staggering from these trials are the modest to minimal impact observed in the placebo arms that include supervised diet and activity interventions. Even in these active intervention comparator arms, after nearly year and a half of supervised activity and diet modifications, patients only experienced modest reductions in body weight - 68 weeks of diet and exercise for 2.4% of body weight. In contrast, the same interventions combined with a GLP-1RA showed an impressive 15% loss of body weight.^14^ Across the phase III trials of the GLP-1RAs, semaglutide, liraglutide, and tirzepatide, the mean weight loss in the placebo arms was 1.6%-5.7% over 3.2-36.8 months. In contrast, GLP-1RAs showed a considerable 5.7%-21% loss of body weight over 3.2-36.8 months. These are impacts not yet seen in any active weight loss programs outside of total life modification approaches such as “The Biggest Loser” reality show^21^ – especially over nearly a year and a half. While chemoprevention of cancer is not the ideal solution – these next generation weight loss drugs (NGWLD) may be a useful tool in the population-level health to stem the rising tide of obesity-related cancers.

**Table 1.** Summary of Seven Phase III Randomized Controlled Trials That Investigated GLP-1 Receptor Agonists and Weight Loss.

Study	GLP-1 Intervention	Lifestyle Intervention	Duration (Weeks)	No. of Participants		Percent Change in Body Weight	
				Placebo	GLP-1	Placebo	GLP-1
Wilding 2021	Semaglutide 2.4 mg per week	Counseling sessions every 4 weeks to encourage a reduced-calorie diet and increased physical activity.	68	655	1306	−2.6 (−2.4)	−15 (−15)
Wadden 2021	Semaglutide 2.4 mg per week	Low-calorie diet for the first 8 weeks and intensive behavioral therapy (ie, 30 counseling visits).	68	204	407	−6.2 (−5.7)	−17 (−16)
Pi-Sunyer 2015	Liraglutide 3.0 mg per day	Counseling on lifestyle modification.	56	1225	2437	−2.8 (−2.6)	−8.4 (−8.0)
le Roux 2017	Liraglutide 3.0 mg per day	Counseling on lifestyle modification.	160	738	1472	−2.0 (−1.9)	−6.5 (−6.1)
Blackman 2016	Liraglutide 3.0 mg per day	Counseling on diet and physical activity. Daily energy intake of 500 kcal. At least 150 min of physical activity/week.	32	178	175	−1.9 (−1.6)	−6.7 (−5.7)
Kim 2013	Liraglutide 1.8 mg per day	Advised to decrease calorie intake by 500 kcal/day.	14	27	24	−3.3 (−3.7)	−6.8 (−7.7)
Jastreboff 2022	Tirzepatide 5 mg per week	Counseling on diet and physical activity. Advised to decrease calorie intake by 500 kcal/day. At least 150 min of physical activity/week.	72	643	630 for 5 mg group	−3.2 (−3.1)	−15 (−15) for 5 mg group
	Tirzepatide 10 mg per week				636 for 10 mg group		−21 (−20) for 10 mg group
	Tirzepatide 15 mg per week				630 for 15 mg group		−22 (−21) for 15 mg group

Abbreviations: GLP-1: Glucagon-like peptide-1; mg: milligram; No.: number.

### Long-Term Impact on Cancer Outcomes

**Figure 1.**
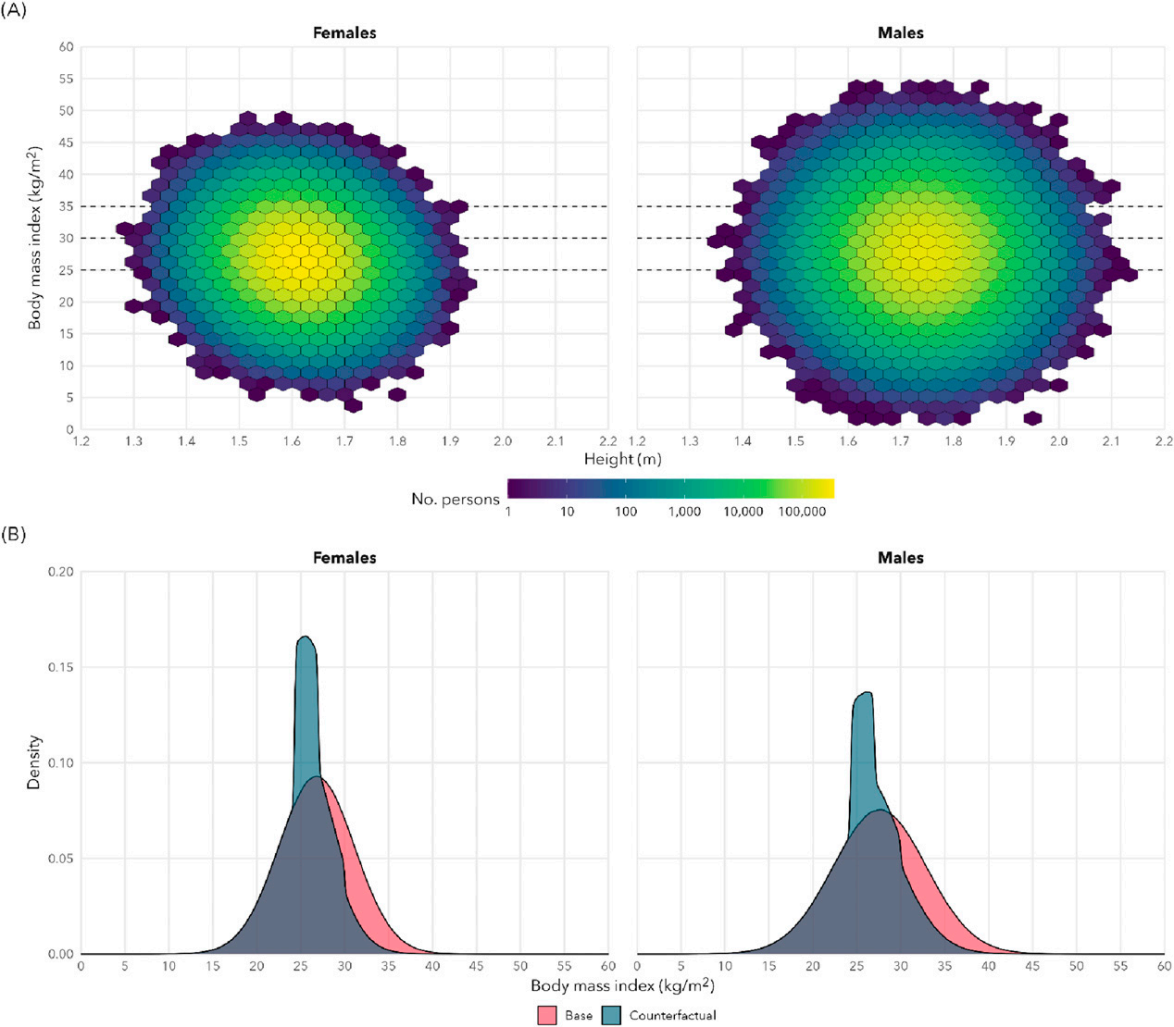
In silico results for the simulated shift in the distribution of body mass index (BMI) for Canadian adults after chemoprevention with GLP-1 receptor agonists in adults with BMI over 27 kg/m^2^. In simulations, a 10% reduction in weight (kg) was applied to everyone with a BMI ≥30 kg/m^2^ and probabilistically to those with BMI between 27 and 30 kg/m^2^.

Our objective was to illustrate the potential of NGWLD in population-level chemoprevention through analyses to estimate the population attributable fractions and potentially avoidable obesity-related cancers in Canada. We conducted analyses using OncoSim, a microsimulation tool developed by Statistics Canada and maintained by the Canadian Partnership Against Cancer (CPAC),^22^ that use population-level data sources from Statistics Canada to evaluate population-level interventions in cancer control. We modeled the simulated effects using a 10% reduction in body weight at the population level as shown in Figure 1. We use 10% as it reflects an approximate effect of the long-term reductions (52 weeks or greater) in weight observed in most of the large phase III trials included in Table 1. We estimated the potential population-level impact of weight loss through chemoprevention with NGWLD in adults with BMI over 27 kg/m^2^. A 10% reduction in body weight was applied to anyone with a BMI ≥30 kg/m^2^, and to 80% of those with BMI between 27 and 30 kg/m^2^. We focused on the number of preventable cancer cases in the Canadian population over the next 25 years. The model assumes a minimal time of 5 years from intervention to reductions in cancer risk with an assumption of short time frame (1 year) from intervention to reduction in BMI.

### Preventable Cancer Cases

**Figure 2.**
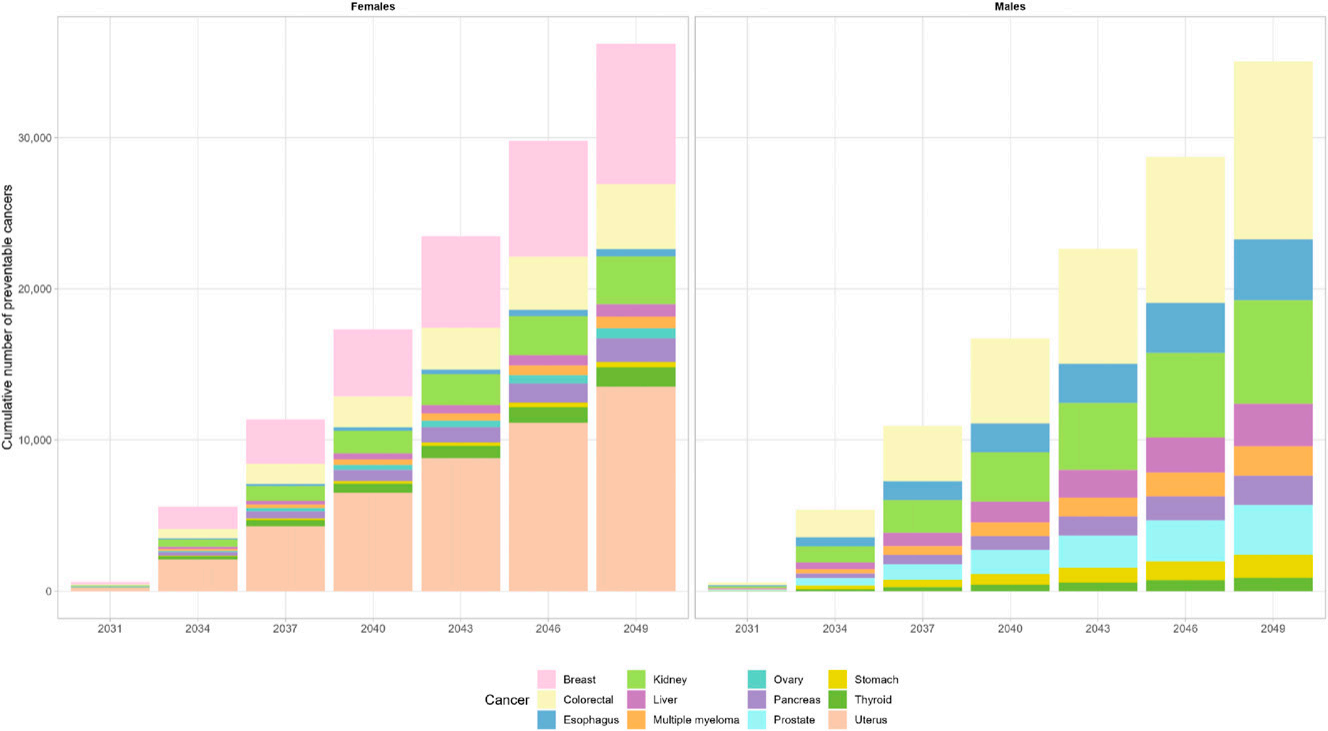
Cumulative number of preventable site-specific obesity-related cancers for females and males through the use of GLP-1 receptor agonists in Canada between 2031 and 2049.

We estimated a total of 71 281 preventable cancer cases by 2049, with 36 235 cases (51%) prevented for females and 35 046 (49%) prevented for males (Figure 2). Among females, the greatest number of preventable cancer cases by 2049 are expected for uterine (13 535), breast (9,313) and colorectal cancers (4,280) (Figure 2). Among males, the greatest number of preventable cancer cases are expected for colorectal (11 767), kidney (6,845) and esophageal (4,042) cancers (Figure 2). Among the 327 254 total projected cancer cases in 2049, 1.3% are estimated to be prevented through intervention with NGWLD. Additionally, we performed sensitivity analyses to evaluate how varying the reduction in body weight to lower (8%) and higher (12%) effectiveness of GLP-1 RA therapy in the real-world would impact the estimates of preventable obesity-related cancers in Canada. For an 8% reduction, we estimated 57 506 preventable cancer cases by 2049, with 29 119 cases (51%) prevented for females and 28 387 (49%) prevented for males (Supplemental Figure 1). For a 12% reduction, we estimated as high as 83 581 preventable cancer cases by 2049, with 42 305 cases (51%) prevented for females and 41 276 (49%) prevented for males (Supplemental Figure 1).

### A Useful Tool in the Toolbox – But Not the Ideal Solution?

While we conducted these analyses to stimulate discussion, at present, we do not support or advocate the use of NGWLD at the population level. Ideally prevention of weight gain with early intervention and awareness is the ideal solution, however, this is proving challenging with overweight and obesity impacting subsequent generations earlier and more often. Many unanswered questions remain regarding the longer-term effectiveness and safety of the agents. The phase III clinical trials commonly reported gastrointestinal side effects, such as nausea, diarrhea, and vomiting, after GLP-1RA treatment.^14-16,20^ Further, a recent cohort study investigated gastrointestinal adverse events in patients using GLP-1RA for weight loss compared to patients using bupropion-naltrexone, a non-GLP-1RA weight loss agent, and reported an increased risk of pancreatitis, gastroparesis, and bowel obstruction for patients using GLP-1RA.^23^ There has been some concern for an increased breast cancer risk with GLP-1 RA therapy due to higher rates of breast neoplasms among liraglutide-treated women compared to placebo groups; however a recent systematic review and meta-analysis of 50 liraglutide clinical trials with 89 000 participants reported no significant increased risk of breast cancer. Most breast cancer cases occurred within 12 months of treatment in women who experienced greater weight loss, which could have facilitated higher mammogram uptake/accuracy and thus, earlier detection of breast masses.^24^ Also of note, early pre-clinical data suggested some potential increase in the risk of rare cancers including thyroid C-cell tumors among users of GLP-1RA; however, since their approval, clinical trials have found no evidence of an increased risk of any cancer with GLP-1 RA therapy.^25^

While NGWLD have shown great promise, long-term challenges in weight maintenance will likely still be a consideration after completion of therapy. After generations where excess body size was beneficial in nature to weather periods of famine or caloric deficits, our bodies have become well adapted to maintaining fat stores. In the weight loss literature, many studies have described a known thermodynamic effect where after considerable weight loss, resting metabolic rates (RMR) slow down to counteract weight loss by reducing energy expenditure. These adaptations make it more difficult for previously overweight individuals to maintain weight loss compared to an individual who was never overweight.^21^ In a study that followed 14 of the original “Biggest Loser” weight loss competitors, the investigators observed that their suppressed RMR that accompanied their initial weight loss through intensive exercise and diet interventions persisted 6 years after the competition ended.^21^ This finding highlights the physiological barriers to maintaining long-term weight loss once an intervention is removed or stopped. Thus, after discontinuation of the NGWLDs, weight rebound may occur without additional individual-level effort or sustaining lower caloric intake. Recent studies have investigated this weight rebound issue in individuals who have discontinued once-weekly semaglutide.^26,27^ In a withdrawal study that investigated the impact of continuing GLP-1 RA therapy vs switching to placebo after 20 weeks (both including lifestyle intervention), 82.4% of the individuals in the placebo group regained weight by week 68.^26^ In contrast, only 15.2% of the individuals who continued semaglutide treatment regained weight.^26^ Wilding et al^27^ reported that 1 year after withdrawal of treatment and lifestyle intervention, individuals regained two-thirds of the weight they had lost over a 68-week treatment period, from a mean weight loss of 17.3% at week 68 to a net loss of 5.6% by week 120.^27^ In the placebo group, individuals had a mean weight loss of only 2% at week 68 and a net loss of .1% at week 120.^27^ Most improvements in cardiometabolic variables also reverted back towards baseline. More recently, analyses of real-world data found a reduction in colorectal cancer risk among users of GLP-1RAs, which was related to the longer-term use of the agents.^28^ These data provide a great deal of support for the underlying theme of chemoprevention in this paper as well as the notion that long-term use may already be occurring in several populations with cancer-related benefit.

It is certain that pharmacologic intervention is not the ideal solution for the obesity-related cancer crisis. However, these agents and subsequent generations provide an additional tool to rapidly reduce body weight and adiposity in populations that have been extremely challenging to reduce weight with standard diet and exercise approaches. Additional research and study into approaches to prevention initial weight gain and maintain long-term effects is needed to avoid bounce back after.

## Supplemental Material

Supplemental Material - Next Generation Weight Loss Drugs for the Prevention of Cancer? Supplemental Material for Next Generation Weight Loss Drugs for the Prevention of Cancer? by Chantelle Carbonell, Mariet Mathew Stephen, Yibing Ruan, Matthew T. Warkentin, and Darren R. Brenner in Cancer Control

## Data Availability

The OncoSim suite of models is available at https://www.partnershipagainstcancer.ca/tools/oncosim/. Access and use are available upon request.
