# Supplementary material for: Next Generation Weight Loss Drugs for the Prevention of Cancer?
Source: Cancer Control. 2024 Mar 22;31:10732748241241158. doi: 10.1177/10732748241241158 (PMC10981222; doi:10.1177/10732748241241158)
Supplement: Supplemental Material - Next Generation Weight Loss Drugs for the Prevention of Cancer? [file sj-pdf-1-ccx-10.1177_10732748241241158.pdf]

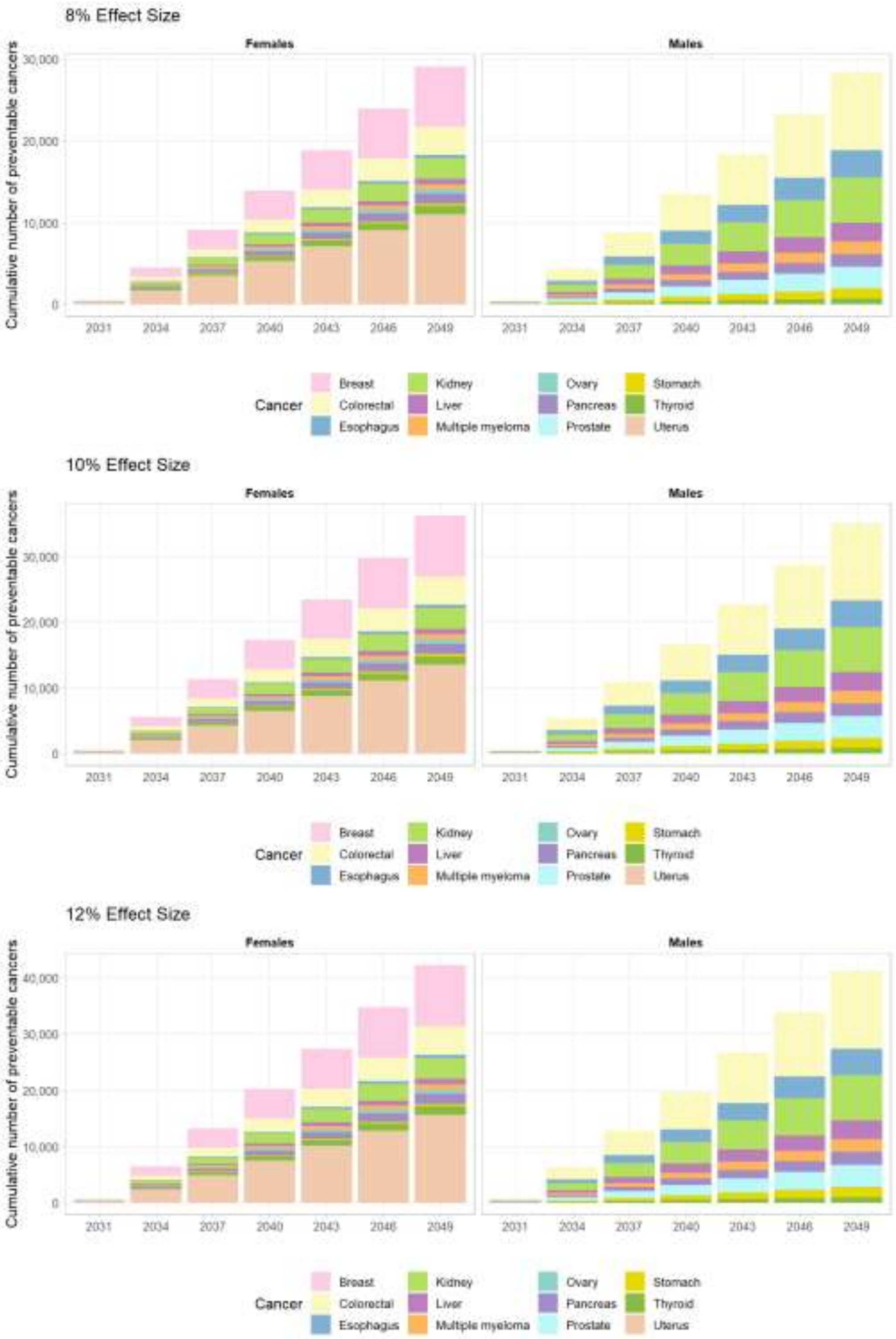

Supplementary Figure 1. Cumulative number of preventable site-specific obesity-related cancers in Canada between 2031 and 2049 for females and males attributable to reduction in body weight (8-12%) through GLP-1 receptor agonist use.
